# Pulmonary embolism in intensive care unit: Predictive factors, clinical manifestations and outcome

**DOI:** 10.4103/1817-1737.62473

**Published:** 2010

**Authors:** Mabrouk Bahloul, Anis Chaari, Hatem Kallel, Leila Abid, Chokri Ben Hamida, Hassen Dammak, Noureddine Rekik, Jameleddine Mnif, Hedi Chelly, Mounir Bouaziz

**Affiliations:** *Department of Intensive Care, Habib Bourguiba University Hospital Sfax, Tunisia*; 1 *Department of Cardiology, Hedi Chaker, University Hospital Sfax, Tunisia*; 2 *Department of Radiology, Habib Bourguiba University Hospital Sfax, Tunisia*

**Keywords:** ICU, predictive factors, prophylactic anticoagulation, pulmonary embolism

## Abstract

**OBJECTIVE:**

To determine predictive factors, clinical and demographics characteristics of patients with pulmonary embolism (PE) in ICU, and to identify factors associated with poor outcome in the hospital and in the ICU.

**METHODS:**

During a four-year prospective study, a medical committee of six ICU physicians prospectively examined all available data for each patient in order to classify patients according to the level of clinical suspicion of pulmonary thromboembolism. During the study periods, all patients admitted to our ICU were classified into four groups. The first group includes all patients with confirmed PE; the second group includes some patients without clinical manifestations of PE; the third group includes patients with suspected and not confirmed PE and the fourth group includes all patients with only deep vein thromboses (DVTs) without suspicion of PE. The diagnosis of PE was confirmed either by a high-probability ventilation/perfusion (V/Q) scan or by a spiral computed tomography (CT) scan showing one or more filling defects in the pulmonary artery or in its branches. The diagnosis was also confirmed by echocardiography when a thrombus in the pulmonary artery was observed.

**RESULTS:**

During the study periods, 4408 patients were admitted in our ICU. The diagnosis of PE was confirmed in 87 patients (1.9%). The mean delay of development of PE was 7.8 ± 9.5 days. On the day of PE diagnosis, clinical examination showed that 50 patients (57.5%) were hypotensive, 63 (72.4%) have SIRS, 15 (17.2%) have clinical manifestations of DVT and 71 (81.6%) have respiratory distress requiring mechanical ventilation. In our study, intravenous unfractionated heparin was used in 81 cases (93.1%) and low molecular weight heparins were used in 4 cases (4.6%). The mean ICU stay was 20.2 ± 25.3 days and the mean hospital stay was 25.5 ± 25 days. The mortality rate in ICU was 47.1% and the in-hospital mortality rate was 52.9%. Multivariate analysis showed that factors associated with a poor prognosis in ICU are the use of norepinephrine and epinephrine. Furthermore, factors associated with in-hospital poor outcome in multivariate analysis were a number of organ failure associated with PE ≥ 3.

Moreover, comparison between patients with and without pe showed that predictive factors of pe are: acute medical illness, the presence of meningeal hemorrhage, the presence of spine fracture, hypoxemia with PaO_2_/FiO_2_ ratio <300 and the absence of pharmacological prevention of venous thromboembolism.

**CONCLUSION:**

Despite the high frequency of DVT in critically ill patients, symptomatic PE remains not frequently observed, because systematic screening is not performed. Pulmonary embolism is associated with a high ICU and in-hospital mortality rate. Predictive factors of PE are acute medical illness, the presence of meningeal hemorrhage, the presence of spine fracture, hypoxemia with PaO_2_/FiO_2_ < 300 and the absence of pharmacological prevention of venous thromboembolism.

Venous thromboembolism (VTE) remains a major challenge in the care of critically ill patients. Subjects in the intensive care unit (ICU) are at high risk for both deep vein thrombosis (DVT) and pulmonary embolism (PE). Pulmonary embolism is a cardiovascular emergency. By occluding the pulmonary arterial bed, it may lead to acute life-threatening condition due to a potentially reversible right ventricular failure. Pulmonary embolism is a difficult diagnosis that may be missed because of a nonspecific clinical presentation. However, early diagnosis is fundamental, since immediate treatment is highly effective. Depending on the clinical presentation, the aim of initial therapy is first to restore a subnormal flow through occluded pulmonary arteries (PA) and second to prevent potentially fatal early recurrences. Both initial treatment and the long-term anticoagulation that is required for secondary prevention must be justified in each patient by the results of an appropriately validated diagnosis strategy.[1]

Despite recent advances in prophylactic, diagnostic and therapeutic modalities, PE is still one of the important causes of in-hospital morbidity and mortality.[2–4] This could be partly due to its nonspecific symptoms and lack of specific physical signs. Not infrequently, these patients require admission in the ICU because of hemodynamic instability or severe hypoxemia. In 2002, 101 000 patients with primary diagnosis of PE were admitted to acute care hospitals in the United States[5] with a case fatality rate of 15% at 3 months. Previous reports have described variable outcome of patients with PE. Reported mortality rates ranged from 8.1% in stable patients to 25% in those with cardiogenic shock and 65% post-cardiopulmonary resuscitation.[6]

The diagnosis of PE is usually suspected by the presence of common symptoms (including breathing difficulties, chest pain on inspiration and palpitations) and clinical signs including low blood oxygen saturation, tachypnea and tachycardia.[1,2] However, in ICU, most of patients require sedation and mechanical ventilation. The clinical manifestations usually observed in this condition (PE) cannot be exhibited by these patients and clinical presentation is usually atypical.[7]

Nevertheless, predictive factors, clinical manifestations and the outcome of PE are rarely studied in the ICUs. The aim of our study is to determine predictive factors, clinical and demographic characteristics of patients with PE in our ICU. Moreover, we aim to define simple predictive factors, which can be considered in routine practice in general ICUs as indicators of poor prognosis in patient with PE.

## Methods

Between January 2005 and December 2008, we prospectively included 87 consecutive patients with a positive diagnosis of pulmonary thromboembolism developed on ICU admission and/or during ICU stay.

In our institution, the diagnosis of PE is usually suspected by the presence of tachypnea, dyspnea, pleuritic chest pain and hemoptysis. However in our ICU, most of the patients required sedation and mechanical ventilation and the diagnosis of PE is usually suspected in patients with un-explicated hypoxemia and/or shock and arterial hypotension. A medical committee of six ICU physicians examined prospectively all available data in order to classify patients according to the importance of clinical suspicion of pulmonary thromboembolism.

The diagnosis of PE is confirmed by a high-probability ventilation/perfusion (V/Q) scan[8] or by spiral computed tomography (CT) scan showing one or more filling defects or obstruction in the pulmonary artery or its branches.[9] The diagnosis was also confirmed when echocardiography showed a direct visualization of a thrombus in the pulmonary artery.[1,2]

The V/Q scan and/or spiral CT scan are performed after correction of hemodynamic instability (using fluid resuscitation and/or catecholamine) and improvement of hypoxemia (using mechanical ventilation, high fraction of O_2_). Massive PE is defined as the presence of hemodynamic instability: arterial hypotension and cardiogenic shock. Arterial hypotension is defined as a systolic arterial pressure <90 mm Hg or a drop in systolic arterial pressure of at least 40 mm Hg for at least 15 min. Shock is manifested by arterial hypotension and by tissue hypoperfusion and hypoxia, including an altered level of consciousness, oliguria, or cool, clammy extremities.[1,2] During the study period, all the patients admitted to our ICU were classified into four groups. The first group includes all patients with confirmed PE. The second group includes 90 patients without clinical manifestations of PE (in this group, pulmonary thromboembolism is not suspected by our medical staff). From this group 90 patients were included in a random way and were analysed in this study.

The third group includes patients with suspected and not confirmed PE (all patients with normal spiral CT scan). The fourth group includes all patients with only DVTs without suspicion of PE.

Our department is a 22-bed medical surgical ICU in a teaching hospital of 510 beds that serves as a first-line medical center for an urban population of one million inhabitants and as a referral center for a larger population coming from south Tunisia. The total number of admissions in our unit is about 1200 per year.

For all included patients, a data entry form was designed to collect demographic, clinical and radiological data on admission and during ICU stay. The patient with a positive diagnosis of PEs, medical files were prospectively reviewed and the following data collected on hospital admission, ICU admission and during ICU stay: age, sex, heart rate, respiratory rate before mechanical ventilation, blood pressure, use of inotropic drugs, the presence of shock, cardiac arrest, fluid intake volume and urinary output. The systemic inflammatory response syndrome (SIRS)[10] was also researched on admission and during ICU stay. Biochemical parameters measured on admission and during the ICU stay are arterial blood gases and acid-base status (pH and HCO_3_), hemoglobin concentration etc.

In our study, the presence of arterial hypoxemia is defined by arterial oxygen saturation in room air ≤92%. In patients receiving mechanical ventilation, arterial hypoxemia is defined as a PaO_2_/FiO_2_ ratio <300.

Moreover, risk factors (immobility, recent surgery within 1 week, comorbid medical conditions, congestive heart failure, chronic obstructive pulmonary disease (COPD), cancer etc.) were also collected.

The use of preventive anticoagulant agents, the delay of development of PE and the clinical manifestations associated with the PE were also recorded for each patient.

On the other hand, chest X-ray findings and arterial blood gas values were recorded. Chest X-rays were analysed by a radiologist who is blinded to the patient's diagnosis. The ECG abnormalities were also recorded.

Due to nonavailability of echocardiography in either ICU or in hospital, only a few patients underwent. In fact, to perform an echocardiography, the patient will need to be transferred to some other hospital. Moreover, almost all patients included in our study have shock associated and/or acute respiratory distress with arterial oxygen tension/FiO_2_ ratio under 300. For these reasons, the echocardiography is rarely performed in our study. For each patient, clinically symptomatic DVTs were researched. Leg ultrasonography also known as *Leg Doppler* is performed when DVT is suspected and when at the same time it is possible to perform Leg ultrasonography in association with spiral CT scan. Estimation of the clinical probability of PE is performed in all patients according to the two scoring systems: the Wells' score[11] and the Geneva revised score,[12] which have been tested prospectively and validated in large clinical trials. Massive PE is defined as the presence of hypotension or shock, whereas submassive PE is defined as stable hemodynamics in the presence of echocardiographic right ventricular (RV) dysfunction based on RV dilatation (end diastolic diameter >30 mm) or hypokinesia or abnormal movement of the interventricular septum with or without tricuspid regurgitation.[13] Therapeutic agents given, either unfractionated heparin alone or thrombolytic agent, were noted. During the ICU stay, all complications were recorded: nosocomial infections,[14] pneumonia, thrombocytopenia, gastrointestinal bleeding, cerebral hemorrhage and hematomas. For each patients, the number of organ failure[14] was calculated on admission and on the day of diagnosis of PE. Moreover, for each patient the severity of illness was estimated with simplified acute physiology score (SAPS II) calculated within 24 h of admission,[15] and according to the APACHE categorization at admission.[16]

The number of patients who died in the ICU and in the hospital was recorded as the primary clinical outcome and patients were grouped accordingly into survivors and nonsurvivors.

Finally, in order to define predictive factors of pulmonary thromboembolism, the group of patients with confirmed PE was compared with the three other groups (without PE, patients with suspected and not confirmed PE and patients with only DVTs without suspicion of PE).

### Statistical analysis

Categorical data are expressed in proportion and subgroups (survival and death; patients with and without PE) are analysed by the Chi-square test.

Continuous variables are expressed as means (± SD) and subgroups evaluated by Student *t*-test. Risk factors are evaluated in univariate analysis and by multivariate analysis by a multiple logistic stepwise regression procedure. Odds ratios are estimated from the b coefficients obtained, with respective 95% confidence intervals (CI 95%).

## Results

### Clinical characteristics and outcome of population with confirmed pulmonary embolism

During the study period, 4408 patients were admitted in our ICU. The diagnosis of PE was confirmed in 87 patients (1.9%), who were all included in this study. In our study the most causes of ICU admission are: traumatic head injury in 15 patients (17.2%), polytrauma in 19 (21.8%), respiratory distress in 28 patients (32.1%), shock in 7 (8%), coma in 8 (9.2%) and post-surgical admission in 7 (8%).

There were 52 males (59.8%) and 35 females (40.2%). The mean age (± SD) was 54.9 ± 20 years with a range of 15 to 87 years. Most patients (73.7%) were older than 40 years.

The clinical presentations of the study group on admission are shown in Table 1. In our study, 67% of patients have a SAPSII score >30 confirming the severity of the patients in the current study. Moreover, 81 patients (93.1%) have one or more organ failure on ICU admission [Table 2]. Neurological failure was the most organ failure observed (54%), followed by respiratory and circulatory failure observed in 52.9% each.

**Table 1:** Patient characteristics at the time of admission to the medical intensive care unit

Parameters	Results (Total number 87) [Range]	%
Age (years)	54.9 ± 20 [15–87]	-
Sex M/F	52/35	
SAPS II	34 ± 14.5 [0–78]	-
ISS	25 ±11.4 [4–51]	-
Class ‘A’ in the APACHE system	49	56.3
Medical patients/Surgical patients	55/32	62/38
HR (beats/min)	101 ± 25.8 [54–203]	-
SBP (mmHg)	117 ± 20 [70–193]	-
Shock	47	54
Use of catecholamine	45	51.7
Use of mechanical ventilation	65	74.7
Hypoxemia (PaO_2_/FiO_2_ < 300)	51	78
Body temperature (°C)	37.5 ± 0.8 [36–40]	-
Glasgow Coma Scale score	11.1 ± 3.8 [3–15]	-
SIRS (Yes/No)	59/28	
Infection (Yes/No)	31/56	
Use of antibiotics (Yes/No)	70/17	
Neurological Deficit	15	
Multi organ failure	81	93.1

**Table 2:** Repartition of patients according to the number of organ failure

Number of organ failure	Number(Total number 87)	Percentage (%)
One	24	27.6
Two	37	42.5
Three	16	18.4
Four or more	4	4.6

Additionally, all patients included in this study, have one or more risk factors and comorbid conditions [Table 3]. In our study, 16 patients (25.4%) did not receive pharmacological prevention of venous thromboembolism because of the presence of contraindications. However, from all patients with no contraindication for anticoagulation, only 76% have received a pharmacological prevention of venous thromboembolism.

**Table 3:** Risk factors and comorbid conditions in all study group

Parameters	Number(Total number 87)	%
Age > 74 (years)	17	19.5
Infection (Yes)	31	35.6
Obesity	15	17
Immobility	87	100
Chronic cardiovascular disease	33	38
Chronic respiratory disease (COPD)	18	20.6
Diabetes	14	16
Connectivity tissue disease	1	1.1
Cancer / Neoplasy	6	6.9
Motor deficit	15	17.2
Fracture of long bones	10	11.5
Spine fracture	11	12.6
Pelvis fracture	4	4.6
Traumatic and non-traumatic brain Injury	44	50.5
Meningeal hemorrhage	24	27.6
Genetic thrombophilia	2	2.3
Blood transfusion	50	57.2
Multi-organ failure	81	93.1

The mean delay of development of PE was 7.8 ± 9.5 days (range 1–60 days). The diagnosis of PE was performed on the day of ICU admission in 9 patients (10.3%). Moreover, 47 patients (54%) developed this complication within five days of ICU admission.

On the day of PE diagnosis, pulmonary auscultation was performed in all patients. It was normal in 54 patients (62%) and showed lung crackles on auscultation of one or both lungs in 22 (25.2%). Table 4 showed the clinical characteristics of all population groups on the day of PE diagnosis.

**Table 4:** Patient characteristics on the day of diagnosis of PE

Parameters	Results(Total number 87)	%
HR (beats/min)	108 ± 21	-
SBP (mmHg)	120 ± 17.5	-
Tachycardia (>90 beats/min)	75	86
Shock	50	57.5
Use of catecholamine	50	57.5
Use of mechanical ventilation	71	74.7
Hypoxemia (PaO_2_/FiO_2_ < 300)	59	83
Chest pain	4	4.5
Hemoptysis	1	1.1
Normal pulmonary auscultation	54	62
SIRS (Yes/No)	63	72.4
Clinical manifestations of deep	15	17.2
vein thrombosis	
Fever (≥ 38°C)	43	49.5
Syncope	4	4.5
Clinical symptoms of right	13	14.9
ventricular dysfunction	

The quantitative plasma *D-Dimer* dosage was performed in 19 patients. A *D-Dimer* value <500 ng/l was observed in 3 patients (15.8%). Ultrasonography of the legs, also known as Leg Doppler, in search of deep venous thrombosis (DVT), was performed in 39 patients (44.8%). It showed DVT only in 15 patients (38.5%). The DVT was proximal in 13 patients and distal in 2 patients.

Chest X-ray was performed in all patients and it was normal in 24 (27.5%) patients. However, the most frequent abnormality noted was bilateral alveolar syndrome in 23 (26.4%) cases followed by lung oligemia in 11 (12.6%) [Table 5].

**Table 5:** Chest X-ray findings of all patients on the day of diagnosis of pulmonary embolism

Findings	Number(Total number 87)	%
Normal	24	27.5
Pleural effusion	7	8
Oligemia	11	12.5
Volume loss/atelectasis	9	10.3
Pulmonary artery enlargement	3	3.4
Elevated hemidiaphragm	5	5.7
Pulmonary infarction	3	3.4
Pulmonary congestion	23	26
Infiltrate	21	24
Pneumothorax	7	8

An electrocardiogram was performed in all patients. The most frequent abnormalities recorded were sinus tachycardia in 61 (70.1%) patients, atrial arrhythmias in 2 (2.2%); complete or incomplete right bundle branch block 16 (18.3%) and T-wave inversion over the right or the left precordial leads (T-wave inversion in leads V2-V3) in 18 (20.6%).

Echocardiography was performed in 17 (19.5%) cases. It showed a normal result in 2 cases, a right ventricular dilatation in 8 and pulmonary artery hypertension in 6. In one case, it showed a direct visualization of a thrombus in the pulmonary artery.

The diagnosis of PE was made by spiral CT in 83 patients (95.4%), a V/Q scan in 3 (3.4%) and by ECG in 1 case.

**Figure 1:**
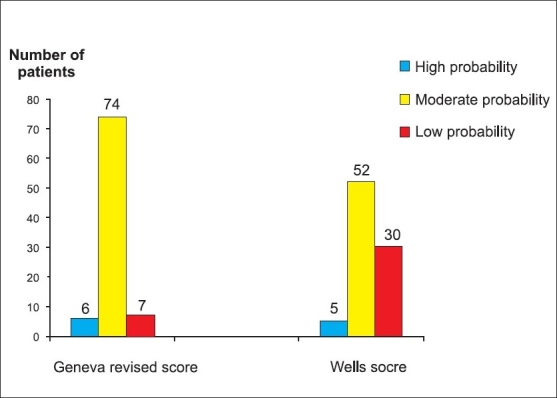
Clinical probability of all patients according to the Wells and Geneva revised score

Estimates of the clinical probability of PE were performed in all patients according to two scoring systems: the Wells' score and the Geneva revised score. Only 5 (5.7%) patients have a high probability according to first score and 6 (6.9%) patients have a high probability according to the second score [Figure 1].

Specific treatment (anticoagulant therapy) is the mainstay of treatment. In our study, 85 patients (97.7%) received parenteral anticoagulants. Intravenous unfractionated heparin was used in 81 cases (93.1%) and low molecular weight heparins were used in 4 cases (4.6%). An inferior vena cava filter was implanted because anticoagulant therapy was contraindicated in 2 cases and/or ineffective in one case. Only one patient received thrombolysis with Streptokinase.

Under anticoagulant therapy, 9 patients (10.5%) have a bleeding complication, including one patient (1.1%), who has an intracranial hemorrhage. Moreover, 16 patients (18.8%) developed thrombopenia.

The mean ICU stay was 20.2 ± 25.3 days (range: 1-203 days) and the mean hospital stay was 25.5 ± 25 days (range: 3-205 days). During their ICU stay, 82 (94%) patients developed one or more organ failure. Cardiovascular failure was the most observed (68.9%). Moreover, 58 (66.6%) developed nosocomial infections. The mortality rate in ICU was 47.1% (41 patients) and the in-hospital mortality rate was at 52.9% (46 patients).

**Figure 2:**
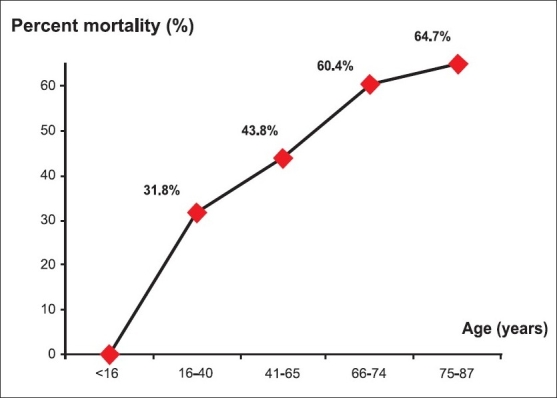
Association between age and mortality in ICU

Compared with survivors, the nonsurvivors were found to be significantly older (61.7 ± 16.4 years vs. 48.8 ± 21.2 years; *P* = 0.002). Moreover, as shown in Figure 2, a good association between age and ICU mortality was found. On the other hand, no significant difference was found in the frequency of cardiovascular, respiratory or renal chronic diseases. Table 6 showed factors associated with a poor outcome in ICU. The multivariate analysis showed that factors associated with a poor prognosis are the use of norepinephrine (*P* = 0.027; Odds ratio (OR)= 6.88; 95% CI = 1.24–38) and the use of epinephrine (*P* = 0.015; OR = 13.9; 95% CI = 1.67–115).

**Table 6:** Factors associated with a poor outcome in ICU on univariate analysis

	Parameters	Survivors	Nonsurvivors	*P*
On ICU admission	Age (Years)	48.8	61.7	0.002
	Sex M/F	25/21	27/14	0.27
	SAPS II score	32.5 ± 15	35.7 ± 13.6	0.3
	Medical patients/Surgical patients	26/20	29/12	0.17
	Number of organ failure ≥3	10.8	36.5	0.004
	Use of catecholamine	41.3%	63.4%	0.03
	Blood urea (mmol/l)	8 ± 4.7	11.4 ± 7.5	0.01
The Day of diagnosis of	Body temperature (°C)	37.9 ± 0.8	37.6 ± 0.7	0.04
pulmonary embolism	Heart rate (beats/min)	107 ± 22	110 ± 20	0.95
	Shock (Yes)	19 (41%)	31 (75.6%)	0.01
	Use of catecholamine	41.3%	75.6%	0.001
	Use of mechanical ventilation	71.7%	92.6%	0.01
	Abnormal chest X ray	60.8%	85.3%	0.01
	pH	7.41 ± 0.06	7.38 ± 0.08	0.04
	Blood urea (mmol/l)	8.6 ± 14	12.8 ± 8.2	0.002
	Blood creatinine (μmol/l)	85 ± 45.9	131.5 ± 126	0.02
	Mean Prothrombinaemia (%)	70.3 ± 11	60.6 ± 20	0.009
	Mean number of organ failure	2	2.7	0.006
	Use of epinephrine (%)	11	41.4	0.001
	Use of norepinephrine (%)	22	53	0.003

In-hospital mortality rate was at 52.9% (46 patients). Table 7 showed factors associated with hospital poor outcome in univariate analysis. Multivariate analysis showed that factors associated with poor outcome are a number of organ failure associated with PE ≥ 3 (*P* = 0.04, OR = 4, 95% CI: 1.05–15).

**Table 7:** Factors associated with a poor hospital outcome on univariate analysis

Parameters	Survivors	Deaths	*P*
Age (Years)	48.8 ± 21.2	61.7 ± 16.4	0.002
Infection (Yes)	24.3%	45.6%	0.03
Body temperature (°C)	37.9 ± 0.9	37.6 ± 0.8	0.04
Shock (the day PE)	43.9%	69.5%	0.01
Use of catecholamine	43.9%	69.5%	0.01
Number of organ failure associated with PE	2 ± 1.2	2.7 ± 0.8	0.006
Blood urea on ICU admission (mmol/l)	7.9 ± 4.8	11.3 ± 7.2	0.01
Blood urea (the day PE) (mmol/l)	8.2 ± 4.9	11.2 ± 7.4	0.03

Day PE= The day of diagnosis of pulmonary embolism

### Predictive factors of pulmonary embolism

As previously shown, during the study period, we have included three other groups of patients:

- The comparison between the PE group and the group without clinical manifestations of PE (90 patients) showed that factors associated with PE in multivariate analysis are: Hypoxemia with PaO_2_/FiO_2_ < 300 (*P* = 0.04, OR: 2.58 and 95% CI: 1.02–6.5), the presence of spine fracture (*P* = 0.002, OR: 32 and 95% CI: 3.5–290) and patients not receiving pharmacological prevention of venous thromboembolism (*P* = 0.006, OR: 3.05 and 95% CI: 1.37–6.80);
- The comparison between the PE group and the group of patients with suspected and not confirmed PE group (44 patients) showed that factors associated with PE in multivariate analysis are: acute medical illness (*P* = 0.026, OR: 3.8 and 95% CI: 1.17–12.3), the presence of meningeal hemorrhage (*P* = 0.034, OR: 4.29 and 95% CI: 1.11–16.5) and the absence of pharmacological prevention of venous thromboembolism (*P* < 0.0001, OR: 23.2 and 95% CI: 6.86–78.5);
- Finally, the comparison between the PE group and patients with only DVTs without suspicion of PE (17 patients) showed that there was no difference between the two groups.

## Discussion

Venous thromboembolism remains a major challenge in critically ill patients. In fact, these patients are at high risk for both deep vein thrombosis (DVT) and pulmonary embolism (PE).[17–20] Critically ill patients commonly develop DVT, with rates that vary from 22% to almost 80%, depending on the patient characteristics.[20] The majority of clots are clinically silent.[19,20] Likewise, the incidence of PE in ICU was poorly described, and systematic screening was not performed.[20] The rate of symptomatic PE ranged from 0.7 to 6%.[20] In our study, the diagnosis of PE was confirmed in 87 (1.9%) out of 4408 patients. The low rate of PE in our study can be explained by the absence of systematic screening in patients who are clinically asymptomatic (false negative).

Population-based studies estimated the risk of thrombosis to be increased two-folds in obese individuals.[21] Chronic lung diseases, cardiovascular diseases and posttraumatic admission were the main comorbid conditions present before the diagnosis of PE in our patients. Moreover, we found in the current study that those patients who do not receive pharmacological prevention of venous thromboembolism developed PE more frequently. Other factors associated with PE were the presence of spine fracture, meningeal hemorrhage and acute medical illness.

Symptoms of PE are sudden-onset of dyspnea and pleuritic chest pain. In ICU, these typical manifestations are rarely observed. In fact, the patients in the ICU here needed intubation, mechanical ventilation and sedation. As a consequence, classical presentation of acute PE may not occur in these patients.

For these reasons, the diagnosis of PE is usually suspected when un-explicated hypoxemia and/or shock and arterial hypotension were observed.

Because of lack of sensitivity and specificity of clinical manifestations, some clinical pre-test probability scores derived from large trials that sought to determine the clinical signs and symptoms predicting the diagnosis of PE were previously validated. In our study, estimates of the clinical probability of PE was performed in all patients according to two scoring systems, which have been tested prospectively and validated in large clinical trials: the Wells' score[11] and the Geneva revised score.[12] These scores may be used to define the probability of PE as low, moderate or high with the prevalence of PE increasing across the three groups. However, in our study according to these scores, only 5 (5.7%) patients have a high probability according to the Wells' score and 6 (6.9%) patients have a high probability according to the Geneva revised score.

When PE is suspected, diagnosis confirmation is needed. In fact, prompt and accurate diagnosis of PE greatly influences patient outcome.[1–4] In our study, the diagnosis of PE was made by spiral CT in 83 patients (95.4%), a V/Q scan in 3 (3.4%) and by ECG in 1 case.

Pulmonary embolism causing hemodynamic instability is termed massive; once it is suspected, a diagnostic plan and supportive measures are essential.[1–3] In our study, all patients received saline infusion. Moreover 50 patients (57%) received catecholamine. The use of catecholamine is associated with poor outcome and multivariate analysis showed that independents factors associated with death are related to the use of epinephrine or norepinephrine. Moreover, OR due to the use of epinephrine may reach 115. This can be explained by the fact that in our ICU, epinephrine is used if reaching hemodynamic objectives with other catecholamine is failed (dobutamine is associated with high dose of norepinephrine).

Oxygen supplement, intubation and mechanical ventilation are instituted when necessary for respiratory failure. When mechanical ventilation is required, care should be taken to limit its adverse hemodynamic effects.[1] In our study, 71 (81.6%) patients required mechanical ventilation. Moreover, the use of mechanical ventilation is associated with a poor outcome.

Anticoagulant treatment plays a pivotal role in the management of patients with PE. Heparin, low molecular weight heparins (such as enoxaparin and dalteparin) or fondaparinux were administered initially.[1–3] The objectives of the initial anticoagulant treatment of PE were to prevent death and recurrent events with low rate of bleeding complications. Considering the high mortality rate in untreated patients, anticoagulant treatment should be considered in patients with suspected PE while waiting definitive diagnostic confirmation.[1–3] Thrombolytic therapy is the first-line treatment in patients with high-risk PE presenting with cardiogenic shock and/or persistent arterial hypotension without absolute contraindications. In our study, intravenous unfractionated heparin was used in 81 cases (93.1%) and low molecular weight heparins were used in 4 cases (4.6%). Moreover, an inferior vena cava filter was implanted because anticoagulant therapy was contraindicated in two cases and/or ineffective in one case. Only one patient received thrombolysis with streptokinase because most patients with cardiogenic shock and/or persistent arterial hypotension have one or more absolute contraindications.

The outcome of patients with PE is quite variable depending primarily on the hemodynamic status and the embolus size. However, other factors have been found in many studies to be useful as prognostic indicators.[1–3] In this study, we described the outcome and the factors associated with poor prognosis of patients with PE admitted to the medical ICU of a teaching hospital in Sfax Tunisia.

The overall in-hospital mortality rate for the 56 patients studied in our center was 47.1%. The MAPPET registry reported an overall mortality rate of 1001 patients with PE to be 29% (14% in the presence of hypotension, 25% in cases of cardiogenic shock and 65% post cardiac arrest).[3] The high mortality rate in our study can be explained by the severity of the patients. In fact, the outcome mentioned here is probably related to the underlying disease rather than PE (93% have multiorgan failure and 54% in shock before PE was developed). Moreover, mean SAPS II score on admission was at 34 ± 14.5 (67% of patients have a SAPSII score >30), mechanical ventilation needed for 74.7% and 81 (93.1%) patients have one or more organ failure on ICU admission. When clinical characteristics of nonsurvivors are compared with those of survivors, older age, acute renal failure, shock and use of catecholamines, use of mechanical ventilation and multiorgan failure are associated with poor outcome. Older age is well known to correlate poorly with survival in PE.[1–3] Moreover, shock, use of catecholamine and/or mechanical ventilation and the presence of multiorgan failure were observed in massive PE usually associated with a poor outcome.[1–3]

There are some limitations to this study that need to be mentioned. Cardiac biomarkers, mainly Troponin and Brain natriuretic peptide, levels are not included in the comparison between survivors and nonsurvivors because they are not available for all patients. In addition, most of our patients were selected on the basis of a positive spiral CT result. Obviously this excluded patients with PE who did not attend hospital and also those who did not have a CT performed in our ICU, either because PE is not considered (biased against atypical cases) or where treatment would not be altered by confirmation of PE. In fact, our study suffers from an absence of systematic screening in patients clinically asymptomatic (false negative). Equally, false negative cases of PE and those who died from PE before a CT scan could be performed would have been excluded. Finally, to date, all studies of PE to date have some inherent bias in their selection of cases, often excluding atypical cases (not diagnosed as PE so not included in the studies) or massive PE where patients died quickly. In summary, this is the first description from a Tunisian ICU of the outcome, risk factors and clinical characteristics of patients with PE admitted to the ICU. The mortality rate in this study is comparable to previously published reports from other parts of the world, despite the fact that most of our patients have significant premorbid conditions and were seriously ill.
